# ABC Cardiol – O Caminho à Frente do Editor-Chefe em 2022 a 2025

**DOI:** 10.36660/abc.20211025

**Published:** 2022-01-01

**Affiliations:** 1 Universidade de São Paulo Faculdade de Medicina Hospital das Clínicas São Paulo SP Brasil Universidade de São Paulo, Faculdade de Medicina, Hospital das Clínicas, Instituto do Coração, São Paulo, SP – Brasil; 2 Hospital do Coração São Paulo SP Brasil Hospital do Coração (HCOR), São Paulo, SP – Brasil; 3 DASA-ALTA São Paulo SP Brasil DASA-ALTA, São Paulo, SP – Brasil

**Keywords:** Cardiologia, Doenças Cardiovasculares, Publicações Periódicas/tendências, Fator de Impacto, Autoria/normas, Políticas Editoriais, Responsabilidade Social

O Arquivos Brasileiros de Cardiologia (ABC Cardiol) é o periódico científico de maior impacto em Cardiologia no Brasil e na América Latina. Em sua história tem tido o papel fundamental de divulgar a produção cientifica nacional em periódico indexado em todas as bases de dados internacionais mais significantes e em língua inglesa. No momento atual constitui-se, portanto, na verdadeira janela da nossa produção científica para o mundo.

As evoluções e resultados positivos que obtivemos nesta gestão atual se apoiam em ações contínuas de gestões anteriores do ABC Cardiol. Aqui aproveito para textualizar meus agradecimentos a todos os Editores-Chefes anteriores, fundamentais na evolução do ABC Cardiol e que permitiu que a gestão atual atingisse resultados positivos, como o do fator de impacto de 2,0^1^ na Journal of Citation Report. A palavra aqui é gratidão a todos os que direta ou indiretamente contribuíram ee torcem para o sucesso do ABC Cardiol e, principalmente, a nossa comunidade científica.^2^

No entanto, os desafios continuam na busca de atingir novos patamares nas missões dos Arquivos Brasileiros de Cardiologia. Nessa jornada de avanços, as melhores ideias vêm da discussão com grupos envolvidos na gestão e da “inteligência coletiva” que Editores-Associados, Assistentes Editoriais, Revisores, membros dos conselhos de publicação da SBC, Editores Anteriores, Diretores da SBC e membros da SBC. Aqui convoco a todos a participarem ativamente do ABC Cardiol e a submeterem seus melhores trabalhos na mais importante revista de Cardiologia do nosso meio.

O que estamos planejando hoje para os próximos 4 anos de gestão, até 2025, estão no texto deste Editorial. Porém novas ideias e ações podem surgir e tudo depende da participação ativa de todos. Este Editor-Chefe tem os ouvidos, mente e coração abertos a sugestão de todos. Contribuam!

Na linha editorial e formato do ABC Cardiol, continuaremos com os minieditoriais convidando os Revisores de artigos a escreverem sua visão sobre o dado original publicado no ABC Cardiol. No ABC Cardiol, quem revisa, escreve minieditoriais!

Estamos estimulando publicações de revisões sistemáticas e metanálises de alta qualidade, o que tem ajudado muito a responder perguntas para as quais estudos clínicos randomizados ainda não estão disponíveis. Temos ainda uma grande missão de divulgar nossos dados populacionais para ajudar nossos gestores públicos a realizarem as melhores decisões em prol da população brasileira.^3-9^ O foco está e continuará nas doenças cardiovasculares sob todos os seus aspectos, incluindo a interdisciplinaridade.

Estudos com dados originais dos programas de pós-graduação de todo o nosso país são a fonte dos artigos de melhor qualidade do ABC Cardiol e sempre avaliamos estes artigos com olhar especial. Programas de pós-graduação das universidades públicas e privadas na área de Cardiologia e das Ciências Cardiovasculares submetam artigos ao ABC Cardiol! Destaque, vídeos, podcasts, repercussão e comentários em nossas páginas totalmente integradas as redes sociais e redes científicas darão a repercussão desejada e muitas vezes superiores a publicações em periódicos internacionais que não darão a devida importância que damos a nossa produção local. A divulgação dos nossos artigos terá prioridade máxima nestes próximos 4 anos e o benefício é de todos, autores, instituições e periódico. Apenas como exemplo, **o novo portal do ABC Cardiol**, que inclui modernas técnicas de procura e permite que *web crawlers* do Google e de outras ferramentas de procura identifiquem nossos artigos publicados e disponibilizem nas buscas efetivadas por usuários nestas ferramentas. Os resultados já são perceptíveis com até 80 mil acessos por mês nas nossas páginas.

Uma nova ação será a indicação de embaixadores do ABC Cardiol nas várias partes do mundo que ajudará a divulgar nossa revista e nossa produção, assim como atrair artigos de alta qualidade destas regiões. Embaixadores da Ásia, Europa, América Latina, Austrália, África e países de língua portuguesa estão programados.

Uma das grandes novidades será a introdução da Figura Central, ou ainda chamada de Resumo Visual ou Resumo Gráfico/Ilustrativo. Esta figura que resume toda a mensagem do artigo científico em uma única figura ou arte gráfica, será distribuída em formatos simples que permitem a rápida difusão pelas mídias sociais e científicas de forma clara e precisa e sempre carregando a referência do nosso artigo. O nome desta arte gráfica em português e inglês para nossa revista ainda não está definido. Mande sua mensagem nos comentários desta página dizendo qual a nomenclatura para esta ilustração você prefere. Participe desta decisão! Ainda no podcast mensal faremos os resumos do número e das novas publicações para os nossos leitores.

A segunda grande novidade planejada para 2023, com transição em 2022, será a publicação continuada, na qual os artigos são publicados de forma imediata após aprovação e diagramação. Isto resultará numa diminuição drástica do tempo entre aprovação e ter o artigo citável com DOI e toda a sua referência para a divulgação dos autores. A meta é reduzir dos atuais aproximados 120 dias para 60 dias ou menos. Os fascículos mensais continuarão a existir, mas serão construídos retrospectivamente. Isto poderá afetar um pouco a paginação do artigo, mas no mundo moderno digital este detalhe perdeu sua importância e vamos nos adaptar aos novos formatos das referencias.

Estamos planejando uma ampliação no nosso corpo de revisores, editores-associados, embaixadores e colaboradores nacionais e internacionais.^10^ Esteja próximo para participar da escrita da história da nossa ciência cardiovascular junto com o ABC Cardiol com 73 anos de história e muito mais pela frente. Venha, faça parte desta história! Estamos de braços abertos a todos da nossa comunidade científica. Planejamos também a ampliação das premiações a revisores e colaboradores assim como tornar a revisão visível através do Publons, ajudando assim a carreira acadêmica de nossos revisores, editores e colaboradores. Ainda em discussão, mas poderemos ainda nesta gestão adotar a publicação dos nomes dos revisores de artigos aprovados, sempre com a aprovação dos revisores e autores.

Nossa internacionalização como periódico continua, com mais um terço das publicações oriundas de centros internacionais. Nossa taxa de aceitação no último ano de aproximadamente 10% apenas dos artigos originais submetidos é um dado que demonstra a qualidade das publicações aprovadas no ABC Cardiol atualmente. Embora isso possa ser um desafio para os autores, a submissão no ABC Cardiol garante uma análise de peer-review (ao menos entre dois editores, no caso da rejeição imediata) e um encaminhamento direto, se apropriado, para as outras revistas da Família ABC e parceiros^11-13^ como: International Journal of Cardiovascular Sciences, ABC Cardiovasular Imaging (revista do DIC), ABC Heart Failure & Cardiomyopahty (revista do DEIC), Revista do DERC, Journal of Transcatheter Interventions (JOTCI da SBHCI), Brazilian Jounal of Cardiovascular Surgery (da Sociedade Brasileira de Cirurgia Cardiovascular). Toda essa oportunidade em uma única submissão! Os editores querem que seu artigo encontre sua casa e morada final o mais rápido possível. E essa integração com a Família ABC aumenta em muita sua chance de atingir este objetivo essencial na carreira acadêmica dos dias de hoje: Publicar! O ABC Cardiol ainda aceita submissões de artigos que já foram publicados em servidores de pre-print reconhecidos pela Scielo e pelo ABC Cardiol como: Scielo pre-prints, MedRxiv, OSFPreprints e Preprints.

O caminho à frente é desafiador, mas estamos prontos para o desafio e vamos crescer muito junto com a nossa comunidade científica que tem um talento fenomenal e que precisa ser mostrado ao mundo.
