# Supplementary material for: ABC Cardiol – O Caminho à Frente do Editor-Chefe em 2022 a 2025
Source: Arq Bras Cardiol. 2022 Jan 1;118(1):1–2. [Article in Portuguese] doi: 10.36660/abc.20211025 (PMC8959046; doi:10.36660/abc.20211025)
Supplement: Translation

# Translation: ABC Cardiol – The Editor-in-Chief-Way Ahead in 2022 to 2025

**Affiliations:** 1 São Paulo SP Brazil Universidade de São Paulo, Faculdade de Medicina, Hospital das Clínicas, Instituto do Coração, São Paulo, SP – Brazil; 2 São Paulo SP Brazil Hospital do Coração (HCOR), São Paulo, SP – Brazil; 3 São Paulo SP Brazil DASA-ALTA, São Paulo, SP – Brazil

**Keywords:** Cardiology, Cardiovascular Diseases, Periodicals Publications/trends, Journal Impact Factor, Authorship/standards, Editorial Policies, Social Responsibility

The Brazilian Archives of Cardiology (ABC Cardiol) is the scientific journal with the greatest impact on Cardiology in Brazil and Latin America. In its history, it has had the fundamental role of disclosing the national scientific production in a journal indexed in all the most significant international databases and in the English language. At present, it is, therefore, the true window of our scientific production to the World.

The positive evolutions and results we have achieved in this current administration are supported by continuous actions from previous ABC Cardiol administrations. Here I take this opportunity to text my thanks to all the previous Editors-in-Chief, who were fundamental in the growth of ABC Cardiol and who allowed the current leadership to achieve positive results, such as the impact factor of 2.0^1^ in the Journal of Citation Report. The word here is gratitude to all those who directly or indirectly contributed and support the success of ABC Cardiol and, especially, our scientific community.^2^

However, challenges continue in the quest to reach new heights in the missions of the Arquivos Brasileiros de Cardiologia. In this journey of advancement, the best ideas come from discussions with groups involved in management and “collective intelligence” that Associate Editors, Editorial Assistants, Reviewers, members of SBC's publishing boards, Previous Editors, SBC Directors and SBC members. Here, I invite everyone to actively participate in ABC Cardiol and submit their best work to the most important Cardiology journal in our country.

What we are planning today for the next 4 years of management, until 2025, are in the text of this Editorial. But new ideas and actions can emerge, and everything depends on the active participation of everyone. This Editor-in-Chief has an open ear, mind, and heart to everyone's suggestion. Contribute!

In the editorial line and format of ABC Cardiol, we will continue with the mini-editorials inviting the Reviewers of articles to write their vision about the original data published on ABC Cardiol. At ABC Cardiol, whoever reviews, writes mini-editorials!

We are encouraging publications of high-quality systematic reviews and meta-analyses, which have greatly helped to answer questions for which randomized controlled trials are not yet available. We also have a great mission to disseminate our populational data to help our public managers to make the best decisions for the benefit of the Brazilian population.^3–9^ The focus is and will continue to be on cardiovascular disease in all its aspects, including interdisciplinarity.

Studies with original data from postgraduate programs throughout our country are the source of the best quality articles on ABC Cardiol and we always evaluate these articles with a special eye. Postgraduate programs from public and private universities in the field of Cardiology and Cardiovascular Sciences submit articles to ABC Cardiol! Highlights, videos, podcasts, reverberations, and comments on our pages, fully integrated with social networks and scientific networks, will give the desired aftermath and often superior to publications in international journals that will not give the due importance that we give to our local production. The dissemination of our articles will have top priority over the next 4 years and the benefit belongs to all, authors, institutions, and journals. Just as an example, the new ABC Cardiol portal, which includes modern search techniques and allows web crawlers from Google and other search tools to identify our published articles and make them available in searches carried out by users in these tools. The results are already noticeable with up to 80 thousand hits per month on our pages.

A new action will be the appointment of ambassadors of ABC Cardiol in various parts of the World that will help to publicize our magazine and our production, as well as attract high-quality articles from these regions. Ambassadors from Asia, Europe, Latin America, Australia, Africa, and Portuguese-speaking countries are scheduled.

One of the big news will be the introduction of the Central Figure, also called Visual Abstract or Graphical/Illustrative Summary. This figure, which summarizes the entire message of the scientific article in a single figure or graphic art, will be distributed in simple formats that allow rapid dissemination through social and scientific media in a clear and precise manner and always carrying the reference of our article. The name of this graphic art in Portuguese and English for our Journal has not yet been defined. Send your message in the comments on this page saying which name for this illustration you prefer. Participate in this decision! Also in the monthly podcast, we will make summaries of the issue and new publications for our readers.

The second big news planned for 2023, with transition in 2022, will be continued publication, in which articles are published immediately after approval and layout. This will result in a drastic reduction in the time between approval and having the article citable with DOI and all its references for publication by the authors. The goal is to reduce the current approximate 120 days to 60 days or less. The monthly issues will continue to exist, but they will be built retrospectively. This may affect the pagination of the article a little, but in the modern digital world this detail has lost its importance and we are going to adapt to the new formats of references.

We are planning to expand our body of reviewers, associate editors, ambassadors and national and international contributors.^10^ Be close by to participate in writing the history of our cardiovascular science alongside ABC Cardiol with 73 years of history and much more to come. Come to be part of this story! We welcome everyone in our scientific community with open arms. We also plan to expand the awards to reviewers and collaborators, as well as make the review visible through Publons, thus helping the academic career of our reviewers, editors, and collaborators. Still under discussion, but we may adopt in this new administration the publication of the names of the reviewers of approved articles, only if the reviewers and authors approve.

The internationalization of our Journal continues, with over a third of publications coming from international centers. Our acceptance rate in the last year of approximately only 10% of the original articles submitted is a fact that demonstrates the quality of publications currently approved by ABC Cardiol. Although this can be a challenge for authors, submission to ABC Cardiol guarantees a peer-review review (at least between two editors in the case of immediate rejection) and direct referral, if appropriate, to the other ABC Family and partners^11–13^ as International Journal of Cardiovascular Sciences, ABC Cardiovascular Imaging (DIC Journal), ABC Heart Failure & Cardiomyopahty (DEIC Journal), DERC Magazine, Journal of Transcatheter Interventions (SBHCI JOTCI), Brazilian Journal of Cardiovascular Surgery (from the Brazilian Society of Cardiovascular Surgery). All these opportunities in a single submission! The Editors want the submitted articles to find their home Journal as quickly as possible. And this integration with the ABC Family greatly increases your chance of achieving this essential objective in today's academic career: To publish! ABC Cardiol even accepts submission of articles that have already been published on pre-print servers recognized by Scielo and ABC Cardiol as Scielo pre-prints, MedRxiv, OSFPreprints, and Preprints.

The way-ahead is challenging, but we are ready for the challenge, and we are going to grow a lot together with our scientific community, which has phenomenal talent, and which needs to be shown to the World.
